# A Commonly Used Chinese Herbal Formula, Shu-Jing-Hwo-Shiee-Tang, Potentiates Anticoagulant Activity of Warfarin in a Rabbit Model

**DOI:** 10.3390/molecules181011712

**Published:** 2013-09-25

**Authors:** Sien-Hung Yang, Chia-Li Yu, Hsing-Yu Chen, Yi-Hsuan Lin

**Affiliations:** 1 School of Traditional Chinese Medicine, College of Medicine, Chang Gung University, Taoyuan 33302, Taiwan; 2 Department of Traditional Chinese Medicine, Chang Gung Memorial Hospital, Taipei 10507, Taiwan; 3 Institute of Molecular Medicine, College of Medicine, National Taiwan University, Taipei 10002, Taiwan; 4 Center for Complementary and Integrative Medicine, National Taiwan University Hospital, Taipei 10048, Taiwan; 5 Graduate Institute of Clinical Medical Sciences, College of Medicine, Chang Gung University, Taoyuan 33302, Taiwan

**Keywords:** herb-drug interaction, Shu-Jing-Hwo-Shiee-Tang, warfarin, Traditional Chinese Medicines, Chinese herbal medicine

## Abstract

*Background*: Drug interactions between traditional Chinese herbal medicines and the anticoagulant warfarin may cause patient harm and are, therefore, important in clinical practice. Our experience in daily practice suggests that prothrombin time (PT) is prolonged when warfarin is used in combination with the Chinese herbal formula Shu-Jing-Hwo-Shiee-Tang (SJHST) commonly used by patients with osteoarthritis. *Objective*: We conducted animal experiments to confirm the effect of SJHST and warfarin on anticoagulant activity. *Methods*: Forty-eight male New Zealand white rabbits were randomized into eight groups of six rabbits. Group A (Control group) was administered normal saline. Group B (Western Medicine group) was administered warfarin 1.5 mg/kg/day. Groups C, D, and E [Traditional Chinese Medicine (TCM) groups] were administered different doses of SJHST (0.5 mg/kg/day, 1 mg/kg/day, and 2 mg/kg/day, respectively). Groups F, G, and H (Combination Therapy groups) were administered warfarin 1.5 mg/kg/day and different doses of SJHST (0.5 mg/kg/day, 1 mg/kg/day, and 2 mg/kg/day, respectively). The total duration of treatment was 14 days. Blood samples were obtained prior to beginning the experiments (day 0) and on day 7, day 14, and day 17 (3 days after discontinuation of the medications). The activated partial thromboplastin time (APTT), PT, and thrombin time (TT) were calculated and compared among the different groups. *Results*: No significant changes were noted in APTT, PT or TT between the control and SJHST-only groups. Significant prolongations of APTT and PTT, but not TT, were observed in the combination groups compared to the warfarin-only group. The enhanced anticoagulant effects returned to normal three days after discontinuation of SJHST treatment. *Conclusions*: We confirmed that the Chinese herb SJHST enhances the anticoagulant effect of warfarin. Although the exact mechanisms of the interaction are unknown, physicians should be aware of the possibility of drug interactions between warfarin and Chinese herbal medicines owing to the increased risk of bleeding.

## 1. Introduction

Traditional Chinese Medicines (TCMs) are widely used in Chinese communities, and their use has recently increased in some Western countries, yet herbal-drug interactions between TCMs and Western Medicines (WMs) are difficult to predict. Warfarin is a commonly used drug for anticoagulant therapy among patients with atrial fibrillation and cardiovascular diseases. The increased risk of bleeding associated with warfarin that is caused by a prolonged prothrombin time (PT) markedly concerns patients, especially older people [1]. Many drugs have reported interactions with warfarin that precipitate the risk of bleeding. Likewise, many herbs have the potential to interact with warfarin and alter pharmacodynamic mechanisms or intrinsic anticoagulant or antiplatelet properties. All of these interactions could result in an increased risk of bleeding [2,3]. This increased risk of bleeding when herbs are combined with warfarin could be caused either by augmenting the anticoagulant effects of the drug, which would result in an increased INR level, or through intrinsic antiplatelet properties, which would not affect the INR [4].

Shu-Jing-Hwo-Shiee-Tang (SJHST), a popular Chinese herbal medicine, has been used for thousands of years in the treatment of degenerative joint diseases, which are prevalent among older patients. SJHST is a mixture of 17 herbs and, thus, the mechanisms of action are complicated and multifaceted. The maximum dose of SJHST is 12 grams per day, according to the pharmacopoeia Wanbinghuichun (“Recovery from All Ailments”, a TCM classic written in the Ming dynasty), and TCM doctors usually prescribe it to be taken in 3–4 separate daily doses. Although there are no published reports regarding coagulopathy caused by interactions between SJHST and warfarin, some patients have exhibited prolonged PT after one to two weeks of using warfarin and SJHST concurrently. PT returned to normal levels approximately one week after SJHST discontinuation in these patients.

The purpose of this study was to explore the possible coagulopathy caused by the concurrent use of SJHST and warfarin in a rabbit model. We aim to prove the existence of an herbal-drug interaction between SJHST and warfarin, and outline parameters for monitoring coagulopathy when using SJHST and warfarin together.

## 2. Results

### 2.1. Warfarin, but not SJHST, Induced Coagulopathy When Used Alone

**Figure 1:**
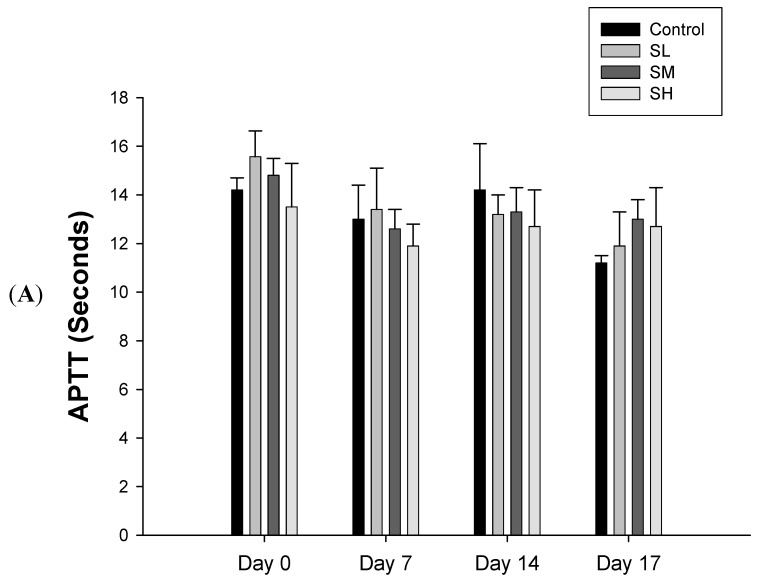

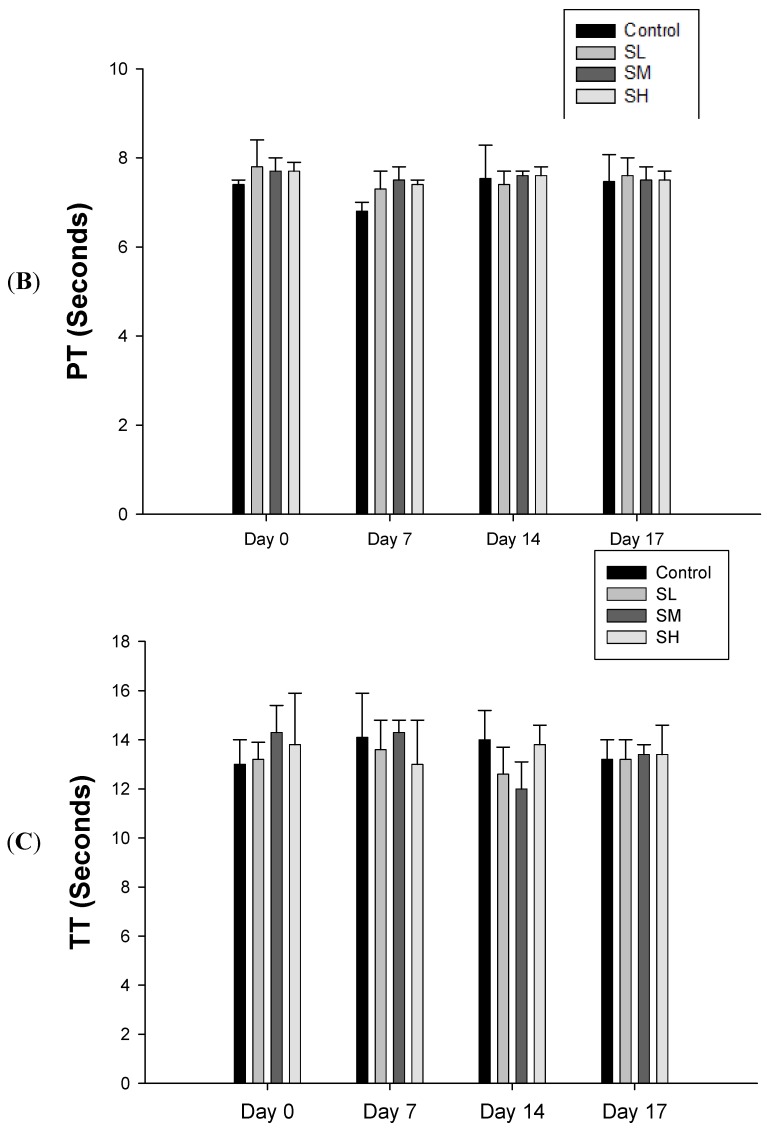
Comparison of activated partial thromboplastin time (APTT) (**A**), prothrombin time (PT) (**B**), and thrombin time (TT) (**C**) in groups of New Zealand White rabbits treated with different doses of Shu-Jing-Hwo-Shiee-Tang (SJHST) only. Control: Treatment with normal saline. SL: Treatment with a low dose (0.5 mg/kg/day) of SJHST. SM: Treatment with a medium dose (1.0 mg/kg/day) of SJHST. SH: Treatment with a high dose (2.0 mg/kg/day) of SJHST. No changes were found in coagulation parameters in rabbits treated with 3 different doses of SJHST for 2 weeks, compared to the control group.

**Figure 2:**
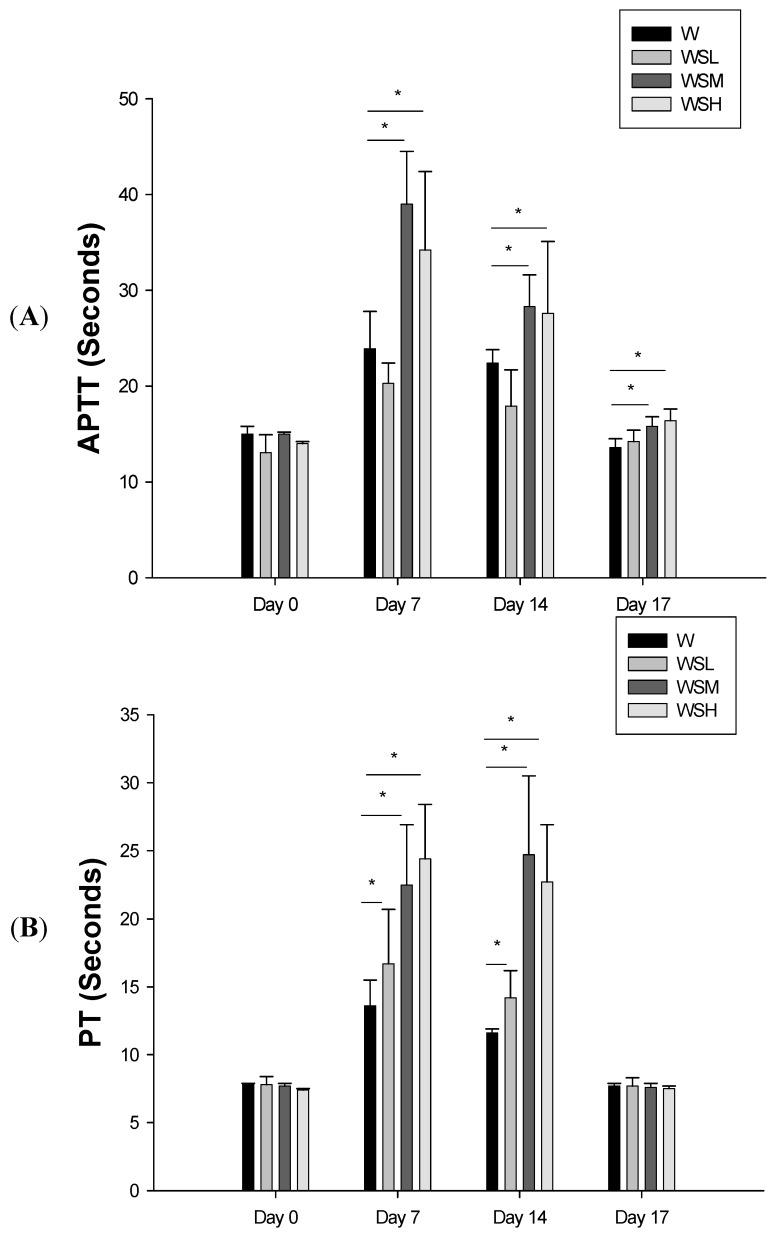

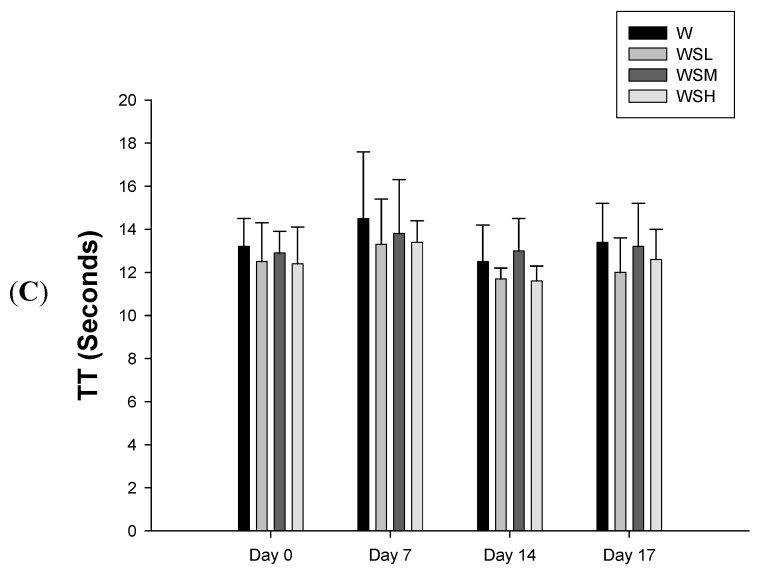
Comparison of activated partial thromboplastin time (APTT) (**A**), prothrombin time (PT) (**B**), and thrombin time (TT) (**C**) in groups of New Zealand White rabbits treated with warfarin and different doses of Shu-Jing-Hwo-Shiee-Tang (SJHST). W: Treatment with warfarin (1.5 mg/kg/day) alone. WSL: Combination treatment with warfarin (1.5 mg/kg/day) and a low dose (0.5 mg/kg/day) of SJHST. WSM: Combination treatment with warfarin (1.5 mg/kg/day) and a medium dose (1.0 mg/kg/day) of SJHST. WSH: Combination treatment with warfarin (1.5 mg/kg/day) and a high dose (2.0 mg/kg/day) of SJHST. APTT and PT were prolonged on day 7 and day 14 in W, WSM and WSH groups. The effects on APTT and PT returned to normal three days after discontinuation of medications in all groups. No significant changes were observed in TT in any of the groups. * p-value < 0.05.

No changes in APTT, PT, or TT were observed in rabbits treated with three different doses of SJHST for two weeks, compared to the control group (Figure 1A–C). In contrast, rabbits treated with warfarin alone demonstrated a significant prolongation of APTT on day 7 (23.9 ± 3.9 s) and day 14 (22.4 ± 1.4 s), compared to day 0 (15.0 ± 0.8 s) (Figure 2A). PT was also longer on day 7 (13.6 ± 1.9 s) and day 14 (11.6 ± 0.3 s), compared to day 0 (7.8 ± 0.1 s) (Figure 2B). No significant changes in TT were observed with warfarin treatment (Figure 2C). The APTT and PT returned to normal three days after the discontinuation of warfarin.

### 2.2. Concurrent Use of Warfarin and SJHST may Induce Coagulopathy

In rabbits treated with warfarin and three different doses of SJHST, we found that the lowest dose of SJHST (0.5 mg/kg/day) increased PT, but not APTT, compared to rabbits administered warfarin alone (Figure 2A,B). With the medium dose of SJHST (1 mg/kg/day), we observed prolonged APTT (15.0 ± 0.2 s on day 0 *vs.* 39.0 ± 5.5 s on day 7and 28.8 ± 3.3 s on day 14) (Figure 2A) and PT (7.7 ± 0.2 s on day 0 *vs.* 22.5 ± 4.4 s on day 7 and 24.7 ± 5.8 s on day 14) (Figure 2B). Similar influences on PT and APTT were also observed with the highest-dose SJHST group (2 mg/kg/day). APTT was prolonged (14.0 ± 0.2 s on day 0 *vs.* 34.2.0 ± 8.2 s on day 7 and 27.6 ± 7.5 s on day 14), as was PT (7.7 ± 0.1 s on day 0 *vs.* 24.4 ± 4.0 s on day 7 and 22.7 ± 4.2 s on day 14) (Figure 2A,B). The prolonged PT and APTT returned to normal three days after the discontinuation of oral drugs. No changes in TT were observed in any of the groups (Figure 2C). No skin bruising or other signs of bleeding were noted in any of the rabbits during the course of this study.

## 3. Discussion

The key finding of this study was that the concurrent use of SJHST and warfarin may potentiate bleeding events by increasing PT and APTT. Three original findings were observed in this animal study. First, no anticoagulant effects were observed at any dose of SJHST alone. Second, when SJHST and warfarin were administered together, APTT and PT were significantly prolonged in the medium-dose group (1 mg/kg) and the high-dose group (2 mg/kg), but not in the low-dose group (0.5 mg/kg). Third, effects of SJHST and warfarin on APTT and PT returned to the normal range within three days after discontinuation of the medications. These findings substantiated our previous observations that some patients suffered from skin bruises when they were taking combination treatment with warfarin and SJHST. 

It is unclear why SJHST enhances the anticoagulant effects of warfarin despite no anticoagulation effect of SJHST alone. The accumulation of coumarin, which belongs to the same family of chemical compounds as warfarin, may be one of the probable mechanisms for the accentuated anticoagulant effects of warfarin. *Angelicae* *sinensis* [5], *Angelica dahurica* [6], *Glycyrrhiza uralensis* [7], and *Zingiber officinale* [8], which are all components of SJHST, are reported to contain chemical compounds from the coumarin-family. Specifically, osthole, psoralen and bergapten are coumarin derivatives contained in *Angelicae sinensis* that could enhance the anticoagulant effects of warfarin [9]. Additionally, the increased risk of bleeding was noted in previous studies based on animal models and case reports [5,6,10,11].

The hypothesis of a coumarin overdose was supported by the longer PT and APTT values in rabbits treated with the highest dose of SJHST, but not with the lowest dose. This effect was observed only when SJHST, especially in higher doses, was used with warfarin. Warfarin is the key factor to prolonged PT and APTT and no significant changes in PT or APTT were observed when SJHST was used alone in this study (Figure 1A–C). Since only four herbs contained in SJHST had coumarin, the insufficient concentration of warfarin family chemical compounds may be the probable reason why SJHST itself had no effects on coagulation. Further, other than coumarin accumulation, the glycosides in another constituent, *Paeonia lactiflora* Pall, are reported to have heparin-like effects and, therefore, it may be responsible for prolonged APTT when using SJHST [12]. On the other hand, SJHST may inhibit intrinsic coagulation pathways, since a dose-dependent relationship was not found with APTT. Chemical compounds, which inhibit disseminated intra-vascular coagulopathy in a similar manner to Tanshinone IIA, may be responsible for this finding [13]. Further study is still needed to clarify these mechanisms of action.

Interference in substrates of cytochrome P450s (CYPs) or P-glycoprotein with enzyme induction or inhibition and/or drug transporters are all potential mechanisms of anti-coagulation effects [14,15]. The influences of herbs on CYPs are not uncommon. Although there is no supporting evidence that cytochrome P450s inhibitors are contained in SJHST currently, more data may be needed to clarify this potential mechanism. Additionally, genetic variations may also be considered, since variations in CYP2C9 and VKORC1 are related to a lower tolerance of chemical compounds in the coumarin family [16]. Therefore, a detailed medical and drug history should be assessed before prescribing SJHST and consideration of genetic variations may be warranted.

Anti-platelet effect may be another factor for bleeding tendency in addition to anti-coagulation effect, since skin bruising is found when concurrently using SJHST and warfarin. Antiplatelet effect, which is highly related to skin bruising, has been found in many constituents of SJHST. For example, isoliquiritigenin in *Glycyrrhiza uralensis* displayed antiplatelet effects in an *in vitro* study, hesperidin in *Citrus reticulate* displayed antiplatelet effects via inhibition of phosphorylation of phospholipase C-gamma 2 and cyclo-oxygenase-1, and Z-ligustilide in *Angelica sinesis* inhibits platelet aggregation [17,18,19]. These effects should also be considered when using SJHST, since aspirin is also extensively used among older people and may participate in an herb-drug interaction; bleeding events may become much more serious once both platelet and coagulation functions are inhibited.

## 4. Experimental

### 4.1. Animals

Male New Zealand white rabbits (body weight 1.8–2.4 kg) were obtained from the Laboratory Animal Center of the Chang Gung Memorial Hospital. Rabbits were maintained in an air-conditioned room at a controlled temperature of 24 ± 2 °C and humidity of 55 ± 15%, with a regulated 12-h light/dark cycle. The study was approved by the Animal Committee of Chang Gung Memorial Hospital.

### 4.2. Medications

The manufacturing process of SJHST begins with decoction and separation of the decoction liquid through a sieve separator. Next, the filtrate is concentrated and the excipients are added during the granulation process. After granulation is complete, the samples are sent for composition analysis by HPLC. The concentration of two essential chemical compounds of SJHST, hesperidin and paeoniflorin, were clearly recognizable when compared to the standard sample (Appendix 1). Only samples that were confirmed by HPLC to meet the pharmacopoeial specifications were used in the present study. We purchased SJHST from Chung Song Zong Pharmaceutical Co., Ltd (Kaohsiung, Taiwan; batch number: EG629-006). Table 1 lists the chemical composition of SJHST used in this study.

**Table 1:** Composition and Preparation of Shu-Jing-Hwo-Shiee-Tang (SJHST).

Latin name	Plant part	Ratio of composition
*Angelica sinensis* (Oliv.) Diels.	root	2
*Ligusticum chuanxiong* Hort.	Stem and root	1
*Paeonia lactiflora* Pall.	root	2.5
*Atractylodes lancea* (Thunb.) DC. or *Atractylodes chinensis*	rhizome	2
*Poria cocos* (Schw.) Wolf	Fungus	1
*Glycyrrhiza uralensis* Fisch.	Root	1
*Rehmannia glutinosa* (Gaertn.) DC.	Root	2
*Clematis chinensis* Osbeck	Root	2
*Zingiber officinale* Rosc	Root	3
*Prunus persica (L.)* Batsch	seed	2
*Cyathula officinalis* Kuan	root	2
*Stephania tetrandra* S. Moore	root	1
*Gentiana manshurica* Kitag*, Gentiana scabra* Bge*.*, *Gentiana triflora* Pall., or *Gentiana regescens* Franch.	root	1
*Angelica dahurica* Benth. et Hood. F or *Angelica dahurica* Benth. et Hook. F. var. formosana Shan et Yuan	root	1
*Citrus reticulate* Blanco	fruit	2
*Notopterygium incisum* Ting. ex H. T. Chang	Stem and root	1
*Saposhnikovia divariata* (Turcz.) Schischk.	Root	1

Warfarin was purchased from Orion Co., Ltd. (Espoo, Finland) and the anesthetic ketamine was purchased from Nang Kung Pharmaceutical Co., Ltd. (Taipei, Taiwan). The muscle relaxant xylazine was purchased from Bayer Co., Ltd. (Projensdorfer, Germany). To avoid possible missing doses and prevent injury to the rabbits due to panic, warfarin and SJHST were both administered by the oral route under anesthesia. A combination of ketamine (12.5 mg/kg) and xylazine (5 mg/kg) was administered in a ratio of 1:1 via intramuscular injection in the posterior leg 5 min before administering the oral medications.

### 4.3. Procedures

Forty-eight rabbits were randomized into eight groups, with six rabbits in each group. Group A (control group) was administered normal saline. Group B (WM group) was administered warfarin 1.5 mg/kg/day. Groups C, D, and E (TCM groups) were administered different doses of SJHST (0.5 mg/kg/day, 1 mg/kg/day, and 2 mg/kg/day, respectively). Groups F, G, and H (combination treatment groups) were administered warfarin 1.5 mg/kg/day and different doses of SJHST (0.5 mg/kg/day, 1 mg/kg/day, and 2 mg/kg/day, respectively). Each group was administered the medications in a single daily dose. The duration of treatment was 14 days. The doses used in this study were calculated according to guidelines established by the United States Food and Drug Administration [20].

### 4.4. Measurement of Coagulation Profile

Blood samples were collected from auricular arteries of fasting animals. A total of 2.5 mL of blood was collected from each animal in sodium citrate blood collection tubes. Samples were obtained prior to experimentation (day 0) and on day 7, day 14 and day 17 (3 days after discontinuation of the medication). The activated partial thromboplastin time (APTT), PT, and thrombin time (TT) were calculated by the Sysmex CA-1500 system (Sysmex; Kobe, Japan).

### 4.5. Statistical Analysis

Results represent the mean ± standard deviation. The data were assessed by the non-parametric Wilcoxon’s signed-rank test to compare the differences before and after treatment in the same group. The Kruskal-Wallis test was used to compare the differences among experimental groups. A value of *p* < 0.05 indicated statistical significance.

## 5. Conclusions

Chinese herbal medicines are often believed to have fewer side effects than WMs and, therefore, they are extensively used alone and in combination with WM. However, potential herbal-drug interactions may exist. This study revealed a potential increased risk of bleeding when larger doses of SJHST are used with warfarin. SJHST is commonly used for degenerative joint diseases and warfarin is commonly used for cardiovascular disease, and thus both of these drugs are commonly used by older patients. Possible herbal-drug interactions should be emphasized by TCM doctors when prescribing anticoagulant therapy to patients. Closer monitoring PT and APTT and risk of bleeding is warranted if patients use warfarin and SJHST together.

## Figures and Tables

**Appendix 1:**
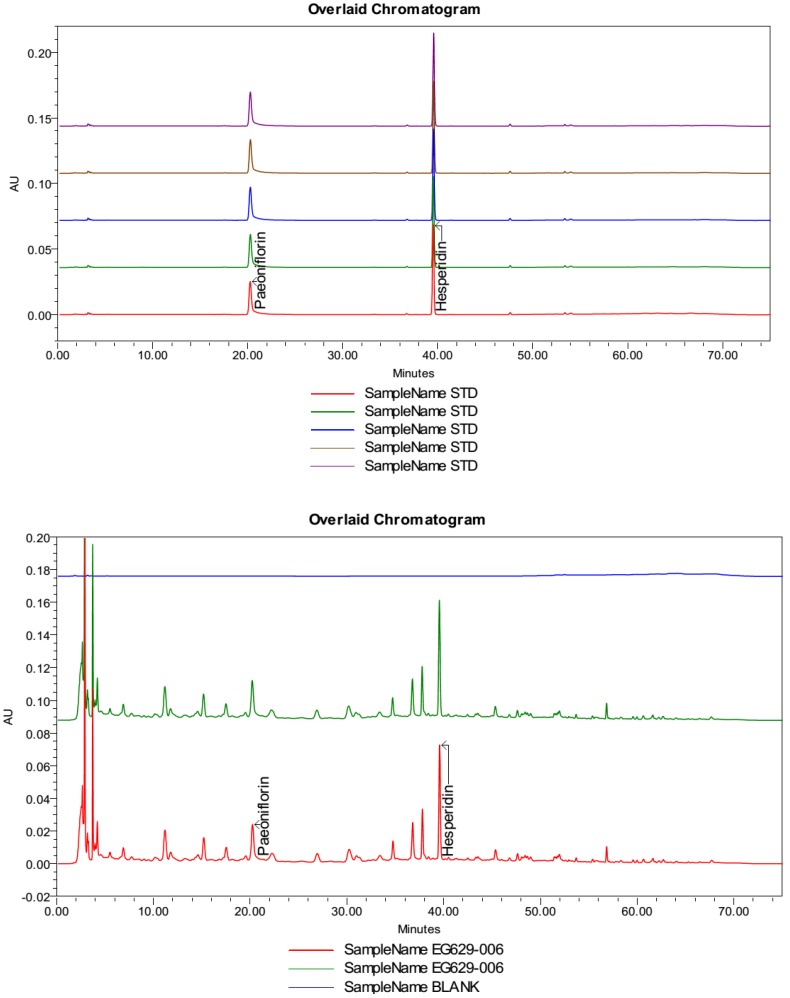
HPLC data of Shu-Jing-Hwo-Shiee-Tang (SJHST) (Upper: standard sample; Lower: SJHST). Different traces indicate different wavelength and the existence of hesperidin and paeoniflorin in both panels proves authenticity of SJHST.

## Supplementary Materials

Supplementary File 1

## References

[1] Almadi M.A., Barkun A., Brophy J. (2011). Antiplatelet and anticoagulant therapy in patients with gastrointestinal bleeding: An 86-year-old woman with peptic ulcer disease. JAMA.

[2] Tsai H.H., Lin H.W., Lu Y.H., Chen Y.L., Mahady G.B. (2013). A review of potential harmful interactions between anticoagulant/antiplatelet agents and Chinese herbal medicines. PLoS One.

[3] Nutescu E.A., Shapiro N.L., Ibrahim S., West P. (2006). Warfarin and its interactions with foods, herbs and other dietary supplements. Expert Opin. Drug Saf..

[4] Samuels N. (2005). Herbal remedies and anticoagulant therapy. Thromb. Haemost..

[5] Lo A.C., Chan K., Yeung J.H., Woo K.S. (1995). Danggui (Angelica sinensis) affects the pharmacodynamics but not the pharmacokinetics of warfarin in rabbits. Eur. J. Drug Metab. Pharmacokinet..

[6] Chen Y., Fan G., Chen B., Xie Y., Wu H., Wu Y., Yan C., Wang J. (2006). Separation and quantitative analysis of coumarin compounds from *Angelica dahurica* (Fisch. ex Hoffm) Benth. et Hook. f by pressurized capillary electrochromatography. J. Pharm. Biomed. Anal..

[7] Mu Y., Zhang J., Zhang S., Zhou H.H., Toma D., Ren S., Huang L., Yaramus M., Baum A., Venkataramanan R., Xie W. (2006). Traditional Chinese medicines Wu Wei Zi (*Schisandra chinensis Baill*) and Gan Cao (*Glycyrrhiza uralensis* Fisch) activate pregnane X receptor and increase warfarin clearance in rats. J. Pharmacol. Exp. Ther..

[8] Shalansky S., Lynd L., Richardson K., Ingaszewski A., Kerr C. (2007). Risk of warfarin-related bleeding events and supratherapeutic international normalized ratios associated with complementary and alternative medicine: A longitudinal analysis. Pharmacotherapy.

[9] Heck A.M., DeWitt B.A., Lukes A.L. (2000). Potential interactions between alternative therapies and warfarin. Am. J. Health Syst. Pharm..

[10] Page R.L., Lawrence J.D. (1999). Potentiation of warfarin by dong quai. Pharmacotherapy.

[11] Lesho E.P., Saullo L., Udvari-Nagy S. (2004). A 76-year-old woman with erratic anticoagulation. Cleve. Clin. J. Med..

[12] Liapina L.A., Ammosova Ia M., Novikov V.S., Osipova N.N., Smolina T., Pastorova V.E., Uspenskaia M.S., Liapin G. (1997). The nature of an anticoagulant isolated from peonies in the central zone of Russia. Izv. Akad. Nauk. Ser. Biol..

[13] Wu L.C., Lin X., Sun H. (2012). Tanshinone IIA protects rabbits against LPS-induced disseminated intravascular coagulation (DIC). Acta Pharmacol. Sin..

[14] Chen X.W., Sneed K.B., Pan S.Y., Cao C., Kanwar J.R., Chew H., Zhou S.F. (2012). Herb-drug interactions and mechanistic and clinical considerations. Curr. Drug Metab..

[15] Shi S., Klotz U. (2012). Drug interactions with herbal medicines. Clin. Pharmacokinet..

[16] Verhoef T.I., Redekop W.K., Daly A.K., van Schie R.M., de Boer A., Maitland-van der Zee A.H. (2013). Pharmacogenetic-guided dosing of coumarin anticoagulants: Algorithms for warfarin, acenocoumarol and phenprocoumon. Br. J. Clin. Pharmacol..

[17] Zhan C., Yang J. (2006). Protective effects of isoliquiritigenin in transient middle cerebral artery occlusion-induced focal cerebral ischemia in rats. Pharmacol. Res..

[18] Tawata M., Aida K., Noguchi T., Ozaki Y., Kume S., Sasaki H., Chin M., Onaya T. (1992). Anti-platelet action of isoliquiritigenin, an aldose reductase inhibitor in licorice. Eur. J. Pharmacol..

[19] Jin Y.R., Han X.H., Zhang Y.H., Lee J.J., Lim Y., Chung J.H., Yun Y.P. (2007). Antiplatelet activity of hesperetin, a bioflavonoid, is mainly mediated by inhibition of PLC-gamma2 phosphorylation and cyclooxygenase-1 activity. Atherosclerosis.

[20] U.S. Department of Health and Human Services, Food and Drug Administration, Center for Drug Evaluation and Research CDER Guidance for Industry: Estimating the Maximum Safe Starting Dose in Initial Clinical Trials for Therapeutics in Adult Healthy Volunteers. http://www.fda.gov/downloads/Drugs/GuidanceComplianceRegulatoryInformation/Guidances/ucm078932.pdf.

