# Supplementary material for: A Commonly Used Chinese Herbal Formula, Shu-Jing-Hwo-Shiee-Tang, Potentiates Anticoagulant Activity of Warfarin in a Rabbit Model
Source: Molecules. 2013 Sep 25;18(10):11712–23. doi: 10.3390/molecules181011712 (PMC6270155; doi:10.3390/molecules181011712)
Supplement: Supplementary File 1 [file molecules-18-11712-s001.pdf]

BioMed Proofreading LLC (Since 2003)

You write it, we polish it.

info@biomedproofreading.com
www.biomedproofreading.com

August 30, 2013

To Whom It May Concern:

This letter confirms that the manuscript entitled *A Commonly Used Chinese Herbal Formula, Shu-Jing-Hwo-Shiee-Tang, Potentiates Anticoagulant Activity of Warfarin in a Rabbit Model* by Dr. Yang et al has been copyedited by native English speakers with a related biomedical background in ***BioMed Proofreading LLC***.

Sincerely,

BioMed Proofreading Services U.S.A.

BioMed Proofreading, LLC

www.biomedproofreading.com

info@biomedproofreading.com

BioMed Proofreading LLC, is a leading English-language copy-editing company founded in 2003 in the U.S.A. Our focus is proofreading and editing of biomedical manuscripts, proposals and grant applications. We comprised medical and life science professionals, with extensive experience as authors and referees for peer-reviewed biomedical journals. During the past eight years, we have provided services for scientists throughout the world, and we are proud to have earned a stellar reputation. As an English editing company we have been evaluated and recommended by ***Brazilian Society of Zoology, Journal of Human Kinetics*** (Poland), ***Journal of Epidemiology (Japan)***, ***Phycology Research Group*** (Czech Republic), ***Chinese Medical Journal***, ***Brazilian Journal of Microbiology*** and ***Brazilian Journal of Medical and Biological Research***.

Crown Centre, Suite 60093, 5005 Rockside Road, Cleveland, Ohio 44131, USA.
Tel: (216)280-4487; Fax: (216)373-5499.
